# Effect of Quorum Sensing Molecule Farnesol on Mixed Biofilms of *Candida albicans* and *Staphylococcus aureus*

**DOI:** 10.3390/antibiotics12030441

**Published:** 2023-02-23

**Authors:** Barbora Gaálová-Radochová, Samuel Kendra, Luisa Jordao, Laura Kursawe, Judith Kikhney, Annette Moter, Helena Bujdáková

**Affiliations:** 1 Department of Microbiology and Virology, Faculty of Natural Sciences, Comenius University in Bratislava, Ilkovičova 6, 842 15 Bratislava, Slovakia; 2 Department of Environmental Health, Research and Development Unit, National Institute of Health Dr. Ricardo Jorge (INSA), Av. Padre Cruz, 1649-016 Lisboa, Portugal; 3 Biofilmcenter, Institute of Microbiology, Infectious Diseases and Immunology, Charité—Universitätsmedizin Berlin, Hindenburgdamm 30, 12203 Berlin, Germany; 4 MoKi Analytics GmbH, Charité-Universitätsmedizin Berlin, Hindenburdamm 30, 12203 Berlin, Germany; 5 Moter Diagnostics, Marienplatz 9, 12207 Berlin, Germany

**Keywords:** methicillin-resistant/sensitive *S. aureus*, biofilms of *C. albicans*-MSSA/MRSA, resistance, farnesol, enhancing effect, cell viability, microscopy

## Abstract

The natural bioactive molecule farnesol (FAR) is widely studied mainly for its antibiofilm and antimicrobial properties. In addition, it increases the effectiveness of some antimicrobial substances, which makes it interesting for the development of combined therapy. In the present work, the effect of FAR either alone or in combination with oxacillin (OXA) on mixed biofilms formed by clinically relevant pathogens, *Candida albicans* and *Staphylococcus aureus*, was studied. *S. aureus* isolates used for biofilm formation originated from blood cultures and central venous catheters (CVC) were characterized in terms of antimicrobial resistance. The minimal biofilm inhibitory concentration (MBIC_50_) for FAR of 48 h mixed biofilms formed by the *C. albicans* and methicillin-sensitive *S. aureus* (MSSA) was determined to be 125 μM, and for the mixed biofilms with methicillin-resistant *S. aureus* (MRSA) was determined to be 250 μM. Treatment of mixed biofilms with OXA (2 mg/mL) showed ≤4% inhibition; however, the combination of OXA (2 mg/mL) and FAR (300 μM) resulted in 80% inhibition of biofilms. In addition, planktonic cells of *S. aureus* exhibited an increased susceptibility to OXA, cefoxitin and kanamycin in the presence of FAR (150 and 300 μM). Scanning electron microscopy (SEM) micrographs confirmed patchy biofilm and lack of candidal hyphae in the samples treated with FAR and FAR/OXA in comparison to control and mixed biofilms treated only with OXA. Intriguingly, in a pilot experiment using fluorescence in situ hybridization (FISH), considerable differences in activity (as indicated by ribosome content) of staphylococcal cells were detected. While the activity rate of the staphylococci in mixed biofilms treated with FAR was high, no FISH-positive signal for staphylococcal cells was found in the biofilm treated with FAR/OXA.

## 1. Introduction

*Candida albicans* represents one of the clinically most important opportunistic pathogens, usually surviving in mixed biofilms. Most often, *Candida* coexists with a grampositive bacterium *Staphylococcus aureus* [1]. Among resistant strains, methicillin-resistant *S. aureus* (MRSA) is highly linked to biofilm production, mostly in bloodstream infections (BSI) [2]. Infections caused by *C. albicans* and *S. aureus* biofilms are usually associated with usage of temporary indwelling medical devices, particularly central venous catheters (CVC), urinary catheters or the application of cardiovascular devices [3,4]. These infections cause high morbidity and mortality, especially in hospitals in intensive care units [5,6]. Several advantages arise from the inter-kingdom relationship that helps to protect the microorganisms from external factors and host immune response [7]. Mixed biofilms formed by *Candida* and *Staphylococcus* are significantly more tolerant to antimicrobial agents than mono-species biofilms. Inhibition of biofilms requires 100 to 1000 folds higher concentrations of antimicrobial agents than planktonic microorganisms and usually requires combined therapy [8,9,10,11,12]. Currently, several systemic and local therapeutic schemes, such as lock solutions, are available for the treatment of infections caused by biofilms [13,14]. Many investigators focus on the development of new antibiofilm and biocompatible materials with incorporated antimicrobial agents, peptides or nanomaterials [15,16,17,18]. Nowadays, there is a strong trend towards the development of hybrid systems of known antimicrobial compounds with more than one mode of action [19].

Quorum sensing (QS) molecules seem to be ideal candidates for developing a combination therapy because of their potential to control pathogens [20,21]. Bacteria and fungi, even higher organisms, naturally produce small diffusible signaling molecules for intra- and inter-species communication. In bacteria, they are mainly involved in the coordination of population density, which is associated with gene regulation related to the production of different virulence factors, such as enzymes or toxins, biofilm formation, the production of antimicrobials or the mediation of conjugation [22]. In fungi, QS molecules mainly affect cell morphology, population growth, virulence control, reproduction, secondary metabolites and pigment production [23]. Tetraprenoid farnesol (FAR) is synthesized as a byproduct of the ergosterol pathway of the polymorphic fungus *C. albicans*. FAR plays a central role in both fungal cells and biofilm physiology in a concentration-dependent manner [24,25]. Concentrations of FAR higher than 100 µM block the morphological shift from the yeast to the hyphal form. This phenomenon is important in mixed biofilms of *C. albicans-S. aureus* because the bacteria preferentially bind into the hyphal filaments through candidal adhesin Als3p, which is not expressed in the yeast form [1]. The antimicrobial and antibiofilm effect of FAR in high concentrations was confirmed also in bacteria [26,27]. In addition, the synergistic effect of FAR with antifungal agents [28,29] and antibiotics [30,31] has been studied. On the other hand, Kong et al. (2017) found that low concentrations of FAR (40–50 µM), considered to be physiological in mixed biofilms of *C. albicans-S. aureus*, resulted in enhanced tolerance of *S. aureus* to antimicrobials [32].

We assume that FAR, as a naturally produced bioactive molecule, could be a promising adjuvant to enhance the effect of antimicrobial agents or for further development of drug delivery materials to prevent biofilm and microbial infection. The aim of the present study is to investigate the inhibiting effect of the combination of FAR and OXA on mixed biofilms formed by *C. albicans* and *S. aureus* with different antibiotic susceptibility profiles.

## 2. Results

### 2.1. Characterization of Strains in Terms of Identification, Resistance and Biofilm Formation

The yeast *C. albicans* SC 5314 [33] and the bacterium *S. aureus* CCM 3953–ATCC 25923 (Czech Collection of Microorganisms, Brno, Czech Republic) were used as standard strains in the experiments. Three clinical isolates of *S. aureus* were used: one from a blood culture and two from CVC. The identity of the microorganisms was verified by the growth on specific cultivating media (CHROM agar Candida, Mannitol Salt Agar). In all of *S. aureus* isolates, the *femA* gene was confirmed [34]. In two of the *S. aureus* isolates, namely DHN 21 528 (further referred to as MRSA1) and L18 (referred to as MRSA2), the methicillin-resistant genotype was confirmed. Standard strain *S. aureus* CCM 3953 (referred to as MSSA1) and the DRA 13 541 isolate (referred to as MSSA2) were evaluated as methicillin-sensitive strains. We observed slower growth of MRSA isolates compared to others (results are summarized in Supplementary Materials Figures S1–S3).

The MRSA isolates were resistant to tested penicillins, cephalosporins, ertapenem, fluoroquinolones, tobramycin, erythromycin and clindamycin. All isolates were susceptible to vancomycin, gentamicin, tetracycline and quinupristin-dalphopristin. The standard strain *S. aureus* MSSA1, was susceptible to all tested antibiotics (Table 1).

All clinical isolates were beta-lactamase producers. Resistance to fluoroquinolones can be associated with the expression of several types of efflux pumps. The *norA*, *norB* and *norC* genes were detected in all of the isolates, but their expression rates were not studied. From the group of aminoglycoside modification enzymes, the aminoglycozide O-nucleozidtransferase (ANTs) was confirmed in both MRSA isolates. Macrolide and lincosamide resistance can be associated with ribosomal binding site modification by methylation or mutation in the 23S rRNA gene encoded by erythromycin ribosome methylases (*erm*) genes. This phenomenon leads to cross-resistance to these antibiotics. In this study, only *ermA* was confirmed in both MRSA isolates. Efflux pumps encoded by the *msrA* and *msrB* genes responsible for pumping macrolides out of the cell were not found in the studied isolates (Table 2; Figures S4 and S5). The standard strain *C. albicans* has been previously characterized in another study by Černáková et al. (2019) [35] and Kucharíková et al. (2011) [36] in terms of antifungal susceptibilities. The strain was susceptible to all tested antifungals used in medical practice, including fluconazole, caspofungin and anidulafungin.

All microbial isolates used in this study showed a moderate ability to form biofilm, when tested as a single species. A slightly weaker biofilm was produced by the two MRSA isolates. This phenomenon was more clearly visible when studying mixed biofilms of *C. albicans*-MRSA (Table 3).

### 2.2. Effect of FAR and the Combination of FAR with Antibiotics to S. aureus Isolates

**Figure 1:**
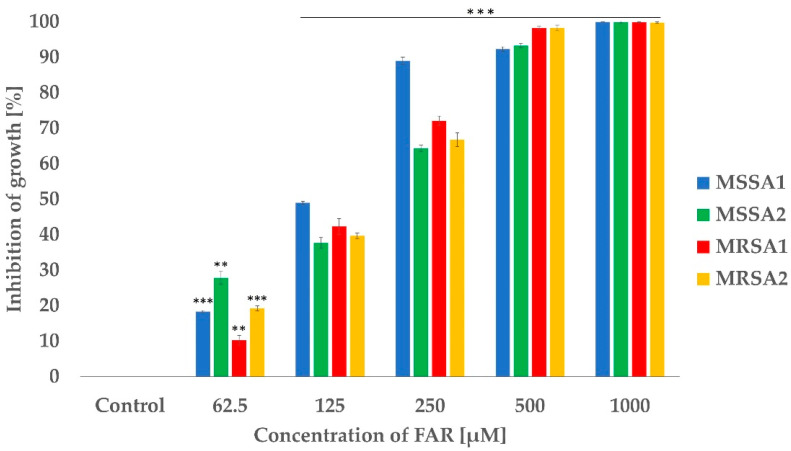
Inhibitory effect of FAR on the planktonic cells of *S. aureus* determined by MIC_50_ in the presence of different concentrations of FAR; the control sample was without FAR. Percentage of growth inhibition was calculated from the OD_570_ values of samples compared to the inhibition of the control sample set to 0%. Data represent the average of 3 independent experiments performed in triplicate. A *p* < 0.01 (**) was considered statistically highly significant; *p* < 0.001 (***) was considered extremely significant.

The effectiveness of FAR was evaluated as MIC_50_, which is the concentration inhibiting 50% of cell growth. At first, planktonic cells of *S. aureus* were tested using the broth microdilution method. The MIC_50_ for the standard strain MSSA1 was 125 µM while for the other isolates, it was one fold higher at 250 µM (Figure 1). The concentration that resulted in 50% inhibition for *C. albicans* was established on 1 mM in a previous study by Černáková et al. (2019) [35].

Two concentrations of FAR (150 and 300 µM) were chosen to monitor its possible potentiating effect in combination with antibiotics on *S. aureus* isolates. Four antibiotics were tested using the E-test: two from the group of beta-lactams, one from the group of aminoglycosides and one from the group of fluoroquinolones. According to previous screening by disc diffusion method, the tested antibiotics were effective against MSSA, while MRSA was resistant to them. The experiment showed that both concentrations of FAR enhanced the effect of oxacillin, cefoxitin and kanamycin; however, the effect was more noticeable in MRSA isolates. Of note, MRSA2 in the presence of FAR (300 µM) reached the MIC for cefoxitin (4 mg/mL). On the other hand, no enhancing effect in the presence of FAR was observed for ciprofloxacin in any of the tested isolates (Table 4). The mode of action of antibiotics can be one of the factors that affects the synergistic effect of FAR with the antibiotics. As FAR acts on the level of cell wall, the effect is most evident using beta-lactams.

### 2.3. Study of the Effect of FAR and the Combination of FAR and OXA on Mono-Species and Mixed Biofilms Formed by Candida albicans-MSSA or MRSA

**Figure 2:**
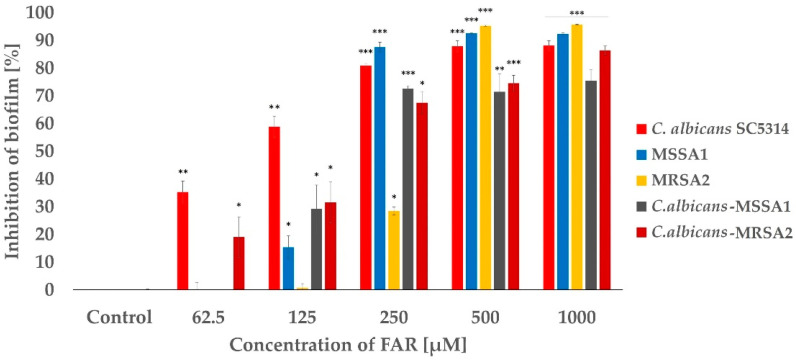
Inhibitory effect of FAR on single-species and mixed biofilms formed by *C. albicans*-MSSA1 and *C. albicans*-MRSA2 determined by MBIC_50_ using the XTT assay. Optical density (OD_490_) of the suspensions was determined after 48 h cultivation of biofilms in the presence of different concentrations of FAR; the control sample was without FAR. Percentage of growth inhibition was calculated from the OD_490_ values of samples compared to the inhibition of the control sample set to 0%. Data represent the average of 3 independent experiments performed in triplicate. A *p* < 0.05 (*) was considered statistically significant; *p* < 0.01 (**) was considered highly significant; *p* < 0.001 (***) was considered extremely significant.

Since resistant and susceptible isolates showed similar characteristics, we selected one representative of each group for further studies. First, the 50% of biofilm inhibition, the MBIC_50_ of FAR, was determined for mono-species and mixed biofilms of *C. albicans*-MSSA1 and *C. albicans*-MRSA2. The MBIC_50_ of FAR for mixed biofilms of *C. albicans*-MSSA1 and *C. albicans*-MRSA2 was established as 125 and 250 µM, respectively (Figure 2). Moreover, inhibition did not differ much at higher concentrations (≥250 µM) in mixed biofilms. Comparing single-species and mixed biofilms, the single-species biofilms were more susceptible to FAR than their mixed counterparts. The only exception was the biofilm of MRSA2, the MBIC_50_ value of which was significantly higher, between concentrations of 250 and 500 µM. Thus, the biofilm formed by MSSA1 was more sensitive than those of the MRSA2 strain. A mono-species *C. albicans* biofilm achieved the MBIC_50_ at a concentration of 125 µM (Figure 2).

**Figure 3:**
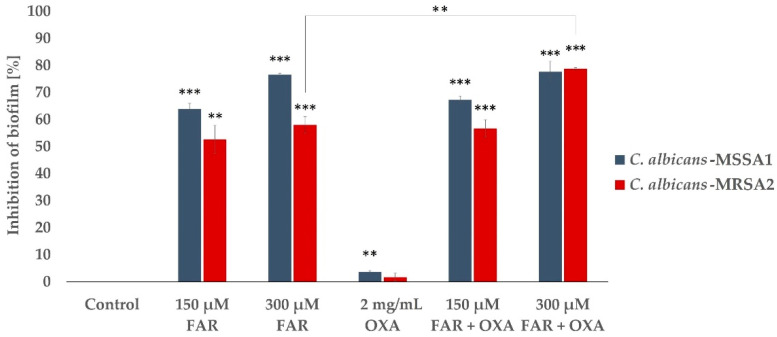
Inhibitory effect of FAR (150 and 300 µM) in combination with OXA (2 mg/mL) on mixed biofilms formed by *C. albicans*-MSSA1 and *C. albicans*-MRSA2 using the XTT assay. Optical density (OD_490_) of suspension was determined after 48 h cultivation of biofilms in the presence of different concentrations of FAR; the control sample was without FAR. Percentage of growth inhibition was calculated from the OD_490_ values of samples compared to the inhibition of the control sample set to 0%. Data represent the average of 3 independent experiments performed in triplicate. A *p* < 0.01 (**) was considered statistically highly significant; *p* < 0.001 (***) was considered extremely significant.

Based on the previous experiment, two concentrations of FAR, 150 and 300 µM, were selected. A concentration of 150 µM was closest to the MBIC_50_ for mixed biofilms of *C. albicans*-*S. aureus*, and the second concentration was chosen to monitor a possible synergistic effect of FAR. The concentration of OXA, 2 mg/mL, represents the MIC for *S. aureus* according to the EUCAST (version 12.0, 2022) [37]. Figure 3 shows the efficacy of FAR and OXA separately and in combination on mixed biofilms, as assessed by the XTT assay. Treatment of mixed biofilms with OXA showed an inhibition of 4% when *C. albicans* was mixed with MSSA and 2% in *C. albicans*-MRSA biofilms. The combination of OXA/FAR (300 μM) represented 80% inhibition of mixed biofilms. However, the expected synergistic effect, particularly in *C. albicans*-MSSA biofilms, was not as significant compared to the biofilm samples treated with FAR alone. Although FAR and the combination of FAR (150 µM)/OXA showed lower efficacy on *C. albicans*-MRSA2 biofilm compared to *C. albicans*-MSSA1 biofilm, the inhibition rate reached the same value of 80% when the concentration of FAR was increased to 300 µM. In single biofilms, the differences in inhibition after FAR treatment compared to FAR/OXA treatment were significantly higher than those observed under the same conditions for mixed biofilms. Moreover, the inhibition rate of OXA (2 mg/L) to the single biofilm formed by MSSA1 isolate showed higher inhibition (≥50%) than when mixed with *C. albicans*.

### 2.4. Microscopic Evaluation of the Effect of FAR and the Combination of FAR with OXA on Mixed Biofilms

**Figure 4:**
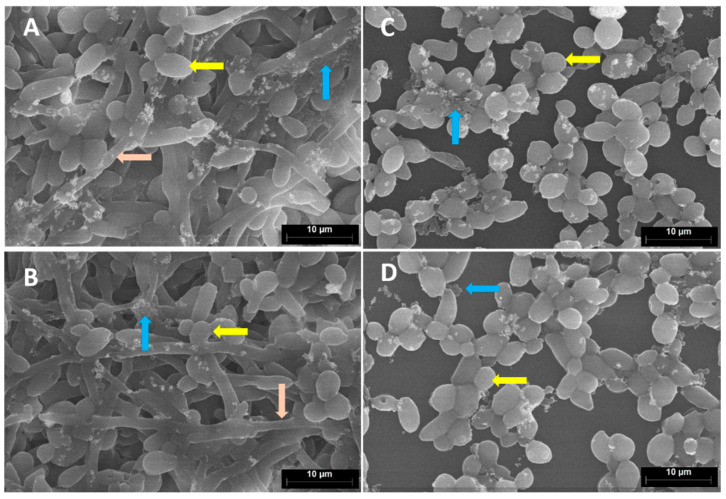
SEM micrographs of mixed biofilms of *C. albicans*-MRSA2 after 48 h of incubation; (**A**) control without treatment, (**B**) treated with OXA (2 mg/mL), (**C**) treated with FAR (300 µM), (**D**) treated with FAR (300 µM)/OXA (2 mg/mL). The scale bars are 10 µm. Yellow arrows show yeast cells, pink arrows show hyphae and blue arrows show staphylococcal cells.

Since the influence of FAR (300 µM) and OXA (2 mg/mL) was similar in both studied biofilms (*C. albicans*-MSSA1 and MRSA2), we decided to choose the *C. albicans*-MRSA2 biofilms for microscopic analysis. SEM was used to characterize the biofilms with regard to biofilm mass, distribution of bacterial and fungal cell forms and the impact of the studied antimicrobial agents on biofilms. SEM micrographs (Figure 4) confirmed damaged biofilm after using FAR (300 µM) and the combination of FAR (300 µM)/OXA (2 mg/mL) in mixed biofilms. Significantly less candidal hyphae were observed in the images of biofilms treated with FAR (Figure 4C,D) in comparison to the control (Figure 4A) and the biofilm treated only with OXA (2 mg/mL) (Figure 4B).

**Figure 5:**
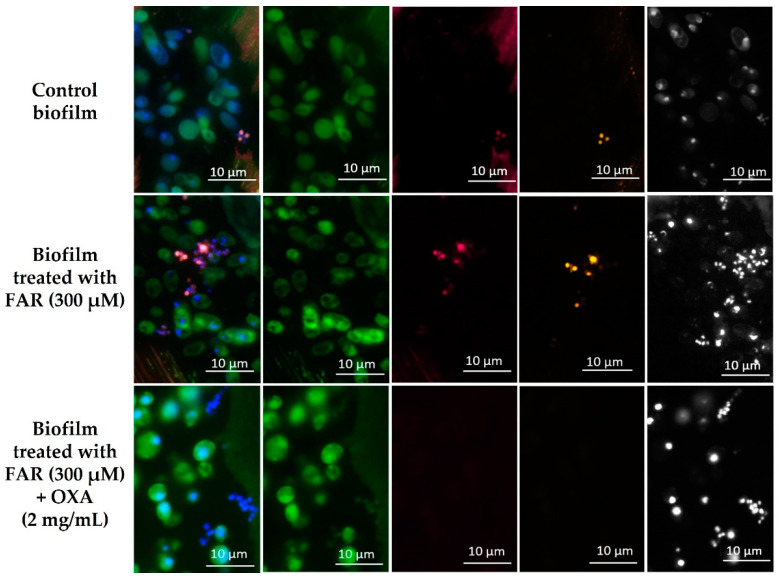
FISH images of mixed biofilms of *C. albicans*-MRSA2 after 12 h of incubation; the upper row represents control biofilm, the middle row represents the biofilm treated with FAR (300 µM) and the lower row represents the biofilm treated with FAR (300 µM)/OXA (2 mg/mL). *Candida* was hybridized with the *Candida*-specific FISH probe CAND10, labeled with FITC (green), almost all bacteria were hybridized with the panbacterial probe EUB338, labeled with Cy5 (magenta), *Staphylococcus* sp. were hybridized with *Staphylococcus* sp.-specific probe (STAPHY), labeled with Cy3 (yellow). Nucleic acids of all cells were stained with DAPI (shown in black-and-white). The first image in each row shows an overlay of all fluorescence channels, followed by CAND10, EUB338, STAPHY and DAPI.

In order to better elucidate the metabolic activity of fungal and bacterial cells based on ribosome content upon treatment with FAR or FAR/OXA, FISH was applied in a pilot study. Representative epifluorescence images are shown in Figure 5. Hybridization was carried out with specific probes for *Candida* sp. (CAND10) [38] and *Staphylococcus* sp. (STAPHY) [39], in combination with the panbacterial probe EUB338 [40]. In the untreated control sample, thin biofilms and patchy groups of microorganisms were detected. All microbial cells (*Candida* and staphylococci) within the biofilm were FISH-positive and therefore presumably metabolically active. A similar result was obtained after 24 h of incubation, however, the biofilm was thicker (upper row). The sample treated with FAR showed thinner biofilms and smaller groups of microorganisms. FISH-positive signals were detected for *C. albicans*; however, part of the staphylococci were FISH-negative. No differences were observed concerning the activity of the microorganism at the two monitored time points (middle row). Microscopic images of mixed biofilms (12 h incubation) treated with the combination of FAR/OXA revealed single cells or small groups of microorganisms. *Candida* showed FISH-positive signals, whereas staphylococcal cells did not show any FISH-signal, indicating their inactive form (lower row).

## 3. Discussion

The yeast *C. albicans* and the bacterium *S. aureus* represent a harmonious combination, often organized in biofilms. In this form, they usually colonize medical devices and become the main cause of healthcare-associated infections (HAIs). According to the ECDC European prevalence study [41], the composite index of antimicrobial resistance of bacteria from HAIs in Slovakia reached 34.8%. By comparison, the overall index of the 29 countries that participated in this survey was 31.6%. On the other hand, the work of Černáková et al. (2022) [42] shows that, compared with bacteria, yeasts are still isolated in a lower number in hospitalized patients in Slovakia. Nonetheless, and critically, there is a high rate of antifungal resistance to at least one antifungal drug (particularly to azoles and 5′-FC—around 82%), which is an important clinical finding. The increased recalcitrance of biofilms to antimicrobial agents and the lack of guidelines for their treatment make these infections riskier and can lead to chronic conditions [43,44]. Microorganisms communicate in biofilms through QS molecules, the use or modification of which could be a promising tool for their eradication [45,46,47].

In the present study, one MSSA and two MRSA isolates were characterized in terms of antimicrobial susceptibility. Both of the MRSA isolates originating from different hospitals showed the same resistance profiles. Based on the source and the phenotype, we can assume that the isolates belong to healthcare-associated MRSA (HA-MRSA). These MRSA isolates commonly harbour SCCmec types I, II, or III, which contain genes that confer resistance to non-beta-lactam antimicrobials [48,49]. In contrast to the community-associated MRSA (CA-MRSA) strains, which are more common but also more virulent, the HA-MRSA tend to cause serious infections such as pneumonia, bacteremia and invasive infections in patients who are exposed to the healthcare setting [50,51]. The third category of MRSA is livestock-associated MRSA (LA-MRSA), which is associated with animal contact [52,53]. In staphylococci, resistance caused by target methylation of ribosomes is relatively widespread. This mechanism confers cross-resistance to macrolides, lincosamides and streptogramins B, known as the MLS_B_ phenotype. One of the methylases, encoded by the *ErmA* gene, was detected in both MRSA isolates. Since the effect of quinupristin-dalphopristin remains active even in methylase-producing staphylococci, testing for streptogramin B would be required to confirm the MLSB phenotype [54]. The MSSA2 also showed multidrug resistance (MDR), which is not rare worldwide [52,55]. However, none of the tested genes for MLS_B_ resistance was detected. Since we did not study other mechanisms, we can only consider enzymatic inactivation, which occurs less frequently than efflux and ribosome-modifying genes in clinical isolates [56].

Biofilm-forming capacity has been described as a virulence factor in *S. aureus* [57]. All three isolates and the standard strain (MSSA1) showed moderate biofilm formation, and in addition, they were *icaA* (gene associated with cell adhesion) positive by PCR. If combined with *C. albicans*, MSSA strains formed stronger biofilms. This was correlated with growth rate (shown in Supplementary Materials Figure S2), where MRSA1 showed the slowest growth compared to other strains. These data are not in agreement with the study of Leshem et al. (2022), where MRSA strains showed higher biofilm-producing capacities when compared to MSSA strains [58]. However, several studies point to high variability between MSSA and MRSA regarding biofilm formation [59,60].

In polymicrobial biofilms, QS represents a key process in cell-cell communication [61]. One of the best-characterized QS molecules is FAR, naturally produced by *C. albicans* [62]. In this study, we showed that for planktonic *S. aureus*, the MIC_50_ of FAR was at concentrations of 125–250 μM (27.75–55.5 μg/mL), which is slightly higher than reported in the literature. Kuroda et al. (2007) reported the MIC for *S. aureus* obtained by broth dilution method reached 125 mg/L and by agar dilution method 2000 mg/L [63]. According to other studies dealing with FAR, the MIC for *S. aureus* ranged from 20 to 80 μg/mL [26,64,65]. The hydrophobic nature of FAR favors its accumulation in the membranes, causing membrane leakage. Inoue et al. (2004) proved significant leakage of K+ ions after exposure to FAR [26]. The study of Kaneko et al. (2011) described the inhibition of 3-hydroxy-3-methylglutaryl (HMG)-CoA reductase as an inhibition mechanism [64]. Kuroda et al. (2007) studied the synergistic effect of FAR with antibiotics and found out that FAR increased beta-lactam susceptibility of MRSA by inhibition of cell wall biosynthesis [63]. In our study, we observed a synergistic effect of FAR with OXA and CEF, a moderate effect with FAR/K and no effect with FAR/CIP tested on *S. aureus* strains. In general, FAR can help antibiotics to penetrate into the cell, but in case of beta-lactams, it interplays with the antibiotic. Therefore, OXA, as a representative of beta-lactams, was selected for further experiments.

The response of biofilms of *S. aureus* strains to FAR was not comparable to planktonic cells. Biofilms, as expected, were more recalcitrant to FAR than to other antimicrobial agents [66]. In addition, the study of Koo et al. (2003) proved that FAR can affect glucan synthesis and consequently reduce the accumulation and biomass of biofilms [67]. The synergistic relationship of *S. aureus* and *C. albicans* results in enhanced recalcitrance of biofilms formed by these pathogens to antimicrobial agents [9,11,68]. In our study, we observed that polymicrobial biofilm tolerated higher concentrations of FAR (≥500 μM) compared to single-species biofilms. The MBIC_50_ for mixed biofilms ranged between 125–250 μM. In single-species *C. albicans* biofilms, the MBIC_50_ was between 62.5–125 μM. Several studies have shown that concentrations higher than 100 μM inhibit mono-species as well as mixed biofilms of *C. albicans*- and *S. aureus* [31,32].

The synergistic effect of OXA (2 mg/mL) was tested in the presence of 150 and 300 μM FAR on mixed biofilms of *C. albicans*-MSSA1 or MRSA2. While OXA alone was not effective enough, 80% of mixed biofilm inhibition was achieved by the combination of 300 μM FAR/OXA. The increased susceptibility of MRSA to OXA in the presence of FAR is preserved in biofilms by the same mechanism described in planktonic cells by Kuroda et al. (2007). FAR inhibits cell wall synthesis through reduction in free C55 lipid carrier, resulting in a subsequent retardation of peptidoglycan monomer precursor transport across the cell membrane. In addition, they proved that FAR affects the secretion and activity of beta-lactamases [63]. Similarly, a reduction in the *S. aureus* population in biofilms was described by Jabra-Rizk et al. (2006) when they studied the combined effect of gentamicin (2.5× MIC) with FAR (200 μM) [31].

In order to examine the architecture and density of mixed biofilms of *C. albicans*-MRSA2 after treatment with FAR (300 μM), OXA (2 mg/mL) and their combination, we used SEM. The untreated biofilm was thicker and harbored hyphae, yeast cells and clusters of staphylococci. A similar result was observed in the samples treated only with OXA, indicating the lack of impact of the antibiotic on mixed biofilm. In samples treated with FAR, no hyphae were present, and microbial communities did not resemble mature biofilms. Ramage et al. (2002) described this phenomenon in *Candida* biofilms when they detected a scant biofilm predominantly composed of yeast cells and pseudohyphae in the presence of 300 μM FAR [24]. Décanis et al. (2011) also detected changes in the cell-wall shape or a visible disconnection between the cell wall and cytoplasm after the addition of FAR [69]. In our study, we observed a slight difference between biofilm treated with FAR and a combination of FAR/OXA, only in the reduced number of staphylococcal cells. Therefore, FISH was applied as a tool for monitoring the activity of the microbial cells within biofilms based on ribosome content. This method allows the visualization and identification of microorganisms by means of specific, 16S rRNA-targeted fluorescent probes based on the amount of ribosomes per cell, which is directly associated with the metabolic activity of microbial cells [70,71]. As a pilot study, the metabolic activity of the microbial cells in mixed biofilms treated with FAR (300 μM) and a combination of FAR (300 μM)/OXA (2 mg/mL) was monitored after 12 and 24 h of treatment. As we wanted to get closer to the clinical scenario, we used polyurethane (PU) as a biofilm carrier material, the material from which CVCs are made. In addition, the MRSA2 strain indeed originated from a CVC infection. Biofilms treated with FAR showed positive FISH signals for *C. albicans* cells and a partially positive signal for *S. aureus*. However, no changes were observed between the two time points studied regarding the activity of the microorganisms. Similar results were published by Koo et al. (2003), who studied 1.33 mM tt-farnesol on biofilms formed by *Streptococcus mutans* and showed only slightly lower numbers of viable cells after treatment [67]. In this study, the FISH-positive signal of *C. albicans* in mixed biofilms treated by the combination of antimicrobial agents remained unchanged. However, no FISH-positive signals and activity of *S. aureus* cells were detected in the sample fixed after 12 h. The results obtained by the XTT test showed that FAR and FAR/OXA have a similar effect on mixed biofilms. First results using FISH suggest that FAR/OXA causes a more pronounced reduction in FISH-signal and microbial activity in the staphylococcal cells. This suggests a synergistic effect of FAR that may sensitize these cells to OXA. More studies are required to determine if these cells are dead or just metabolically inactive in a resting stage. Despite the promising results, one of the main limitations of the study is the inclusion of a small number of isolates. However, the tested isolates were selected according to preliminary results from a collection originating from the hospitals of Slovak Republic. To support the presented results, it could be beneficial to extend the study with isolates from other regions with different characteristics.

## 4. Materials and Methods

### 4.1. Characterization of Microbial Strains

In this work, standard strains of *C. albicans* SC 5314 [33] and *S. aureus* CCM 3953-ATCC 25923 (Czech collection of microorganisms, Brno, CR), and 3 clinical isolates of *S. aureus* were used; DHN 21 528 isolated from a blood culture provided by the HPL laboratories in Bratislava (SK), DRA13 541 isolated from the tip of the CVC of a pediatric patient provided by the University Hospital in Bratislava (SK) and L18 strain acquired from CVC kindly provided by Prof. Lívia Slobodníková, Ph.D. from the Institute of Microbiology, Bratislava (SK). Mueller Hinton (MH) broth or agar was used for bacterial growth, and yeast extract–peptone–dextrose medium (YPD) was used for *Candida* cultivation. All cultivating media were purchased from Biolife, Milan, Italy. Microorganisms were preserved at −20 °C in an appropriate broth supplemented with 60% glycerol.

### 4.2. Antimicrobial Susceptibilities and Resistance Signatures of S. aureus Strains

The susceptibility profiles of the *S. aureus* isolates were determined using the disc diffusion method according to the protocol outlined by the European Committee on Antimicrobial Susceptibility Testing (EUCAST) (version 12.0, 2022) [37]. Briefly, the overnight cultures of bacteria were washed twice in a physiological solution of phosphate-buffered saline (PBS, 137 mM NaCl, 2.7 mM KCl, 10 mM Na_2_HPO_4_, 2 mM KH_2_PO_4_, pH 7.4, all chemicals from AppliChem, Darmstadt, Germany). Bacterial cells were adjusted to a concentration corresponding to 0.5 McFarland Standard turbidity (1.5 × 10^5^ bacteria/mL). A volume of 100 µL of bacterial culture was inoculated onto the MHA and antibiotic discs were distributed on top of the media (Oxacillin 10 µg/mL, Cefotaxime 30 µg, Cefoxitin 30 µg, Ceftazidime 30 µg, Ciprofloxacin 5 µg, Ofloxacin 5 µg, Gentamicin 10 µg, Tobramycin 10 µg, Vancomycin 30 µg, Erythromycin 15 µg and Tetracycline 30 µg). The media were incubated for 24 h at 37 °C. The measured susceptibility values were evaluated according to the EUCAST guidelines (EUCAST, version 12.0, 2022) [37].

BD BBL™ Cefinase™ disks were used for the determination of beta-lactamases production in *S. aureus* according to the manufacturer’s instructions (Becton Dickinson, Canaan, CT, USA). A few colonies were transferred to a disk, and after 1 h of incubation at 37 °C, the color change of the disk was read; yellow and red color represented a positive reaction, and no color change indicated no beta-lactamases production.

Polymerase chain reaction (PCR) was performed for the detection of genes related to different antimicrobial resistance profiles. Oligonucleotide primer sequences and their properties are listed in Table S1. Genomic DNA was isolated with HigherPurity™ Bacterial Genomic DNA Isolation Kit according to the manufacturer’s instructions (CanvaxBiotech, Córdoba, Spain). The total volume of the PCR was 20 μL and consisted of 4 μL 5× FIREPol^®^ Master Mix (Solis BioDyne, Tartu, Estonia), 1 μL (0.01–10 ng/μL) of template DNA, 0.5 μL of 10 pM Forward primer, 0.5 μL of 10 pM reverse primer and 14 μL nuclease-free water. The PCR reaction was performed in an iCycler Thermal Cycler (BIORAD, USA). Nuclease-free water was used as a negative control. PCR programs were used with modifications according to primers described previously: *mecA* [72], *norA*, *norB*, *norC* genes [32], *aph(3′)-IIIa*, *ant(4′)-Ia*, *aac(6′)-Ie/aph(2″)* genes [73], *ermA*, *ermB*, *ermC* genes [74], *msrA* gene [75], *msrB* gene [76]. Visualization of PCR products was performed in 1.5% agarose gel in Tris-borate-EDTA buffer (TBE) with 4 μL of GoodView Nucleic Acid Stain-HGV-II (SBS Genetech, Beijing, China), and the DNA Ladder (Invitrogen, Carlsbad, CA, USA) was used to estimate the length of products. Electrophoresis was performed at 80 V for 90 min (PowerPac™, Bio-Rad Laboratories Inc., Hercules, CA, USA). After separation, DNA fragments were visualized using an UV-Transilluminator MUV 21-312-220 (Major Science, Sea Gull Way Saratoga, CA, USA) at a wavelength of 254 nm.

### 4.3. Biofilm Assay

Determination of the ability of *S. aureus* isolates, *C. albicans* and a combination of *C. albicans-S. aureus* to form biofilm was performed as described by Ramage et al. (2001) [77] and Harriott and Noverr (2009) [9] with modifications. Biofilms were classified based on absorbance (OD_570_) according to Stepanovic et al. (2000) [78]: OD ≤ 0.2—weak biofilm, 0.2 < OD ≤ 0.4—moderate biofilm, 0.4 < OD ≤ 0.6—strong biofilm and OD > 0.6—very strong biofilm. For mono-species biofilms, overnight cultures of *S. aureus* in MHB or *C. albicans* in YPD were prepared. Cells were pelleted by centrifugation and washed twice in PBS. Finally, the pellets were resuspended in an MHB medium with 2% glucose. *C. albicans* was adjusted to a concentration of 2 × 10^6^ cells/mL using a Bürker chamber. The *S. aureus* cell suspension was diluted to OD_570_ = 0.5 corresponding to 1 × 10^8^ cells/mL. One hundred microliters of cultures of *C. albicans* or *S. aureus* were added into a high-adherence 96-well microtitre plate in 3 parallel wells and supplemented with 100 μL of MHB with 2% glucose. After 90 min of static incubation at 37 °C, non-adherent cells of *C. albicans* were removed. Plates with microorganisms were then continuously incubated for 24 h.

For mixed biofilms, both microorganisms, *C. albicans* and *S. aureus*, were added together into the 96-well microtitre plate. One hundred microliters of *C. albicans* and 50 μL of *S. aureus* cultures were prepared in the same way as for mono-species biofilms, and wells were adjusted with MHB with 2% glucose to 200 μL. The plates were statically incubated for 24 h at 37 °C. The quantity of biomass was evaluated using a 0.1% crystal violet solution and measured spectrophotometrically at OD_570_ (Dynex MRX-TC Revelation, Dynex Technologies, Chantilly, VA, USA). Briefly, the biofilms were washed twice in PBS solution and air-dried for 10 min at room temperature (RT). Following that, 110 µL of 0.1% crystal violet solution was added to each sample and incubated for 45 min. The samples were washed three times in distilled water, and then 200 µL of 96% ethanol was added to each the sample and incubated for 45 min. Finally, 110 µL of each sample was transferred into a new well and measured spectrophotometrically at OD_570_ (Dynex MRX-TC Revelation, Dynex Technologies, Chantilly, VA, USA), against ethanol.

### 4.4. Susceptibility Testing of FAR to Planktonic S. aureus

Susceptibility testing was carried out using the microdilution method according to the EUCAST protocol (version 12.0, 2022) [37]. Overnight cultures of *S. aureus* were washed twice in PBS and adjusted to 5 × 10^5^ bacteria/mL in MHB. The stock solution of FAR (75 mM, Sigma-Aldrich, Steinheim, Germany), prepared in 96% ethanol (Centralchem, Banská Bystrica, Slovakia), was diluted in MHB medium to obtain final concentrations in the wells, namely 1000, 500, 250, 125 and 62.5 µM (corresponding to 222, 111, 55.5, 27.75, 13.88 mg/mL). Next, 100 μL of the bacterial cell suspension and 100 μL of FAR at the appropriate concentration were added to the wells. Bacteria without treatment served as a positive control. From each sample, three parallel wells were prepared. The plates were incubated statically for 24 h at 37 °C. The effectiveness of FAR was determined in terms of MIC_50_. The intensity of bacterial growth was measured spectrophotometrically at OD_570_ (Dynex MRX-TC Revelation, Dynex Technologies, Chantilly, VA, USA) against the control with MHB.

### 4.5. Susceptibility Testing of FAR with Antibiotics on Planktonic S. aureus

The susceptibility of FAR in combination with Oxacillin, Cefoxitin, Kanamycin and Ciprofloxacin was tested using E-test strips (Liofilchem, Roseto Degli Abruzzi, Italy). Overnight cultures were washed twice in PBS and adjusted to a concentration corresponding to 1 McFarland Standard turbidity (3 × 10^5^ bacteria/mL) in MHB. One hundred microliters of the bacterial solution was spread onto the MHA and MHA supplemented with 150 and 300 µM FAR. The FAR stock solutions in MHB were prepared as described in Section 4.4. After drying the media with inoculum, antibiotic strips were carefully placed on the agar surface and incubated for 24 h at 37 °C. E-tests were evaluated in terms of MIC and compared to the tables of EUCAST (version 12.0, 2022) [37].

### 4.6. Susceptibility Testing of FAR on Mono-Species and Mixed Biofilms

For determination of the effect of FAR, 48 h old biofilms were grown. Overnight cultures of *S. aureus* were washed twice in PBS and diluted to OD_570_ = 0.5, corresponding to 1 × 10^8^ bacteria/mL in MHB medium supplemented with 2% glucose. One hundred microliters of inoculum and 100 μL of MHB medium with 2% glucose or medium with FAR in the appropriate concentration were added into a 96-well plate. The range of tested FAR concentrations was the same as described in Section 4.4 (62.5, 125, 150, 250, 300, 500, or 1000 µM). After 24 h of incubation, the biofilms were carefully washed in PBS and the old medium was exchanged for fresh medium including the appropriate concentration of FAR. The samples were then incubated for an additional 24 h. The metabolic activity of biofilm samples was measured by the reduction in XTT [2,3-bis(2-methoxy-4-nitro-5-sulfophenyl)-2*H* tetrazolium-5-carboxanilide] as described by Ramage et al. (2001) [77]. Briefly, the biofilms were washed twice in PBS, and then 110 μL of XTT (0.5 mg/mL) in PBS and menadione were added. The samples were incubated in the dark for 90 min at 37 °C. The absorbance was measured at OD_490_ against the XTT with menadione.

To prepare a 48 h old biofilm of *C. albicans*, the overnight culture was washed with PBS twice and adjusted to 4 × 10^6^ cells/mL in MHB with 2% glucose using the Bürker chamber. One hundred microliters of inoculum and 100 μL of MHB with 2% glucose or medium containing FAR at the appropriate concentration were added to a 96-well plate. After 24 h of incubation, the biofilms were carefully washed in PBS and a new medium (with FAR) was added. After 24 h of incubation, the samples were evaluated by the XTT method as described above.

The preparation of 48 h old mixed biofilms of *C. albicans-S. aureus* initially followed the same process as in the case of single-species biofilms. First, the *C. albicans* biofilm was assembled and after 24 h of incubation, 50 μL of *S. aureus* suspension was added to the pre-formed biofilm. MHB with 2% glucose or medium including FAR at the appropriate concentration was adjusted to 200 μL and incubated for 24 h at 37 °C. The metabolic activity of the samples was evaluated using the XTT method.

### 4.7. Susceptibility Testing of FAR/OXA Combination on Mono-Species and Mixed Biofilms

The procedure and conditions for the preparation of 48 h old biofilms for this experiment were the same as described in Section 4.6. Two concentrations of FAR, 150 and 300 µM, were prepared from the stock solutions of FAR (75 mM, Sigma-Aldrich, Steinheim, Germany) as described in Section 4.4. The final concentration of OXA (Sigma-Aldrich, Steinheim, Germany) was 2 mg/mL, representing the MIC for *S. aureus* (EUCAST, version 12.0, 2022). The antimicrobial agents were added at t=0 and after 24 h of the sample’s incubation.

### 4.8. Microscopic Analysis of Mixed Biofilms

For the SEM analysis, 48 h old biofilms of *C. albicans-S. aureus* were prepared in 24-well plates as described in Section 4.6. Biofilms were treated with FAR (300 μM), OXA (2 mg/mL) or with a combination of FAR/OXA. After 48 h of incubation at 37 °C, the biofilms were fixed with 4% paraformaldehyde (Sigma-Aldrich, Steinheim, Germany) in PBS and incubated for 1 h in the dark at RT. The fixative was removed, and the samples were washed twice in PBS for 10 min. Samples were post-fixated with 1% osmium tetroxide (Sigma-Aldrich, Steinheim, Germany) in PBS for 1 h in the dark and on ice. Then, the samples were washed twice in PBS and deionized water for 10 min each at RT. The samples were dehydrated using a serial dilution of ethanol: 25%, 50%, 70% and 95%, each step for 10 min in the dark and on ice. Finally, 100% ethanol was added for 15 min, and this step was repeated one more time. After complete drying, the biofilm samples formed on the bottom of the 24-well plate were carefully cut from the plate using heat. Sputter-coated samples with carbon (20 nm) using a Sputter Coater QISOT ES (Quorum Technologies, Lewes, UK) were mounted on the SEM sample holder with carbon tape and analyzed under an electron microscope, Hitachi S2400 (Hitachi, Tokyo, Japan) using a secondary electron detector.

For FISH analysis, *C. albicans*-MRSA2 biofilms were grown on polyurethane (PU) carriers (VARNISH-PU 2 KW of Isomat S.A., Thessaloniki, Greece) to mimic catheter material. The PU carriers used in this study were prepared according to Dadi et al. (2021b) [79]. The 48 h biofilms were prepared as was described in the Section 4.6. Untreated control, biofilm treated with FAR (300 µM) and biofilm treated with a combination of FAR (300 µM)/OXA (2 mg/mL) were fixed at two time points, 12 h and 24 h after the addition of *S. aureus* to the pre-formed *C. albicans* biofilms. The PU carriers were carefully washed in PBS and fixed in FISH fixation solution FISHopt (MoKi Analytics, Berlin, Germany) overnight. After removal of the fixative, the samples were washed in PBS and dehydrated with 100% acetone for 1 h at 4 °C. Then, the infiltration solution of Technovit^®^ 8100 (Kulzer KmbH, Wehrheim, Germany) was added into the samples and incubated for 10 h at 4 °C. The samples were carefully transferred into Eppendorf tubes filled with the polymerization solution of Technovit^®^ 8100 and allowed to harden at 4 °C. Samples were sectioned into 2 µm sections. Hybridization was carried out with specific FISH probes targeting ribosomes of *C. albicans* (CAND10) [38], labeled with FITC (green), the panbacterial probe EUB338 [40], labeled with Cy5 (magenta) and the *Staphylococcus* sp.-specific probe STAPHY [39], labeled with Cy3 (yellow). The nucleic acid stain DAPI was used to visualize all microbial nucleic acids in the biofilm samples.

### 4.9. Statistical Analysis

Results were evaluated by statistical analysis using a one-way *t*-test. Differences were considered statistically significant at *p* < 0.05 (*), *p* < 0.01 (**) and *p* < 0.001 (***).

## 5. Conclusions

We have demonstrated a sensitizing effect of FAR on planktonic MSSA and MRSA isolates treated with beta-lactams and kanamycin, but not ciprofloxacin. This effect was not significant in mixed biofilms of *C. albicans*-MSSA or MRSA, although staphylococcal cells became inactive after the combined treatment of FAR and OXAllin. Thus, we may conclude that FAR acts on several levels. By blocking hyphae, it prevents the formation of a compact biofilm and, at the same time, increases the sensitivity of MSSA or MRSA to beta-lactam antibiotics. Therefore, FAR could be a promising adjuvant for the treatment of mixed biofilms of *C. albicans*-MSSA as well as MRSA.

## Figures and Tables

**Table 1:** Antimicrobial susceptibility profiles of *S. aureus* isolates.

Antibiotic Group	Antibiotic/Dose	Strain of *S. aureus*			
		MSSA1	MSSA2	MRSA1	MRSA2
Penicillins	Oxacillin 10 µg	S	S	R	R
	Ampicillin 10 µg	S	R	R	R
Cephalosporins	Cefotaxime 30 µg	S	S	R	R
	Cefoxitin 30 µg	S	S	R	R
Carbapenems	Ertapenem 10 µg	S	S	R	R
Glycopeptides	Vancomycin 30 µg	S	S	S	S
Fluoroquinolones	Ciprofloxacin 5 µg	S	S	R	R
	Ofloxacin 5 µg	S	R	R	R
Aminoglycosides	Gentamicin 10 µg	S	S	S	S
	Tobramycin 10 µg	S	S	R	R
Macrolides	Erythromycin 15 µg	S	R	R	R
Lincosamides	Clindamycin 2 µg	S	R	R	R
Streptogramines	Quinupristin/dalphopristin 15 µg	S	S	S	S
Tetracyclines	Tetracycline 30 µg	S	S	S	S

S: susceptible; R: resistant.

**Table 2:** Resistance signatures of *S. aureus* isolates.

Antibiotic Group	Resistance Signatures	*S. aureus* Isolates			
		MSSA1	MSSA2	MRSA1	MRSA2
Beta-lactams	*mecA*	−	−	+	+
	Beta-lactamases production	−	+	+	+
Fluoroquinolones	*norA*	+	+	+	+
	*norB*	+	+	+	+
	*norC*	+	+	+	+
Aminoglycosides	*ant(4′)-Ia*	−	−	+	+
	*aph(3′)-III*	−	−	−	−
	*aac(6′)-aph(2″)*	−	−	−	−
Macrolides, lincosamides and streptogramin B	*ermA*	−	−	+	+
	*ermB*	−	−	−	−
	*ermC*	−	−	−	−
	*msrA*	−	−	−	−
	*msrB*	−	−	−	−

+: present, −: not present.

**Table 3:** The ability of biofilm formation of *S. aureus* isolates, *C. albicans* and the combination of *C. albicans-S. aureus* in mixed biofilms.

Microbial Strains	OD_570_	SD	Biofilm Intensity
MSSA1	0.324	0.034	moderate
MSSA2	0.337	0.012	moderate
MRSA1	0.228	0.01	moderate
MRSA2	0.291	0.013	moderate
*C. albicans*	0.363	0.03	moderate
*C. albicans*-MSSA1	0.413	0.093	strong
*C. albinans*-MSSA2	0.476	0.036	strong
*C. albicans*-MRSA1	0.34	0.03	moderate
*C. albicans*-MRSA2	0.376	0.094	moderate

SD: standard deviation.

**Table 4:** Effect of FAR (150 and 300 µM) on the Antibiotic Susceptibility Profile of *S. aureus* isolates.

		MSSA1	MSSA2	MRSA1	MRSA2
		MIC (mg/mL)			
OXA	Control	0.38	0.75	48	32
	150 µM FAR	0.19	0.5	24	12
	300 µM FAR	0.19	0.38	4	6
FOX	Control MIC	2	3	256	256
	150 µM FAR	1.5	2	96	256
	300 µM FAR	1	2	16	2
K	Control MIC	1.5	3	256	256
	150 µM FAR	1	3	96	256
	300 µM FAR	0.5	0.75	24	48
CIP	Control MIC	0.25	0.19	32	32
	150 µM FAR	0.094	0.094	32	32
	300 µM FAR	0.012	0.032	32	32

OXA—oxacillin, FOX—cefoxitin, K—kanamycin, CIP—ciprofloxacin.

## Data Availability

The data presented in this study are available on https://zenodo.org/record/7595525 (accessed on 1 February 2023).

## Supplementary Materials

The following supporting information can be downloaded at: https://www.mdpi.com/article/10.3390/antibiotics12030441/s1, Figure S1 Specific growth of *C. albicans* SC 5314 on CHROMagar Candida; Figure S2: Phenotype identification and growth curves of *S. aureus* isolates; Figure S3: PCR detection of *mecA* and *femA* genes in isolates of *S. aureus*; Figure S4: PCR detection of *norA,B,C* genes in isolates of *S. aureus*; Figure S5: PCR detection of *ant(4′)-Ia* and *ermA* genes in isolates of *S. aureus*; Table S1: List of oligonucleotide sequences. References [32,34,72,73,74,75,76] are cited in the supplementary materials.
