# Supplementary material for: Effect of Quorum Sensing Molecule Farnesol on Mixed Biofilms of Candida albicans and Staphylococcus aureus
Source: Antibiotics (Basel). 2023 Feb 23;12(3):441. doi: 10.3390/antibiotics12030441 (PMC10044556; doi:10.3390/antibiotics12030441)
Supplement: Supplementary file 1 [file antibiotics-12-00441-s001.zip › antibiotics-2225746-supplementary.pdf]

Supplementary Materials

Effect of Quorum Sensing Molecule Farnesol on Mixed Biofilms of *Candida albicans* and *Staphylococcus aureus*

Barbora Gaálová-Radochová [1,*], Samuel Kendra [1], Luisa Jordao [2], Laura Kursawe [3,4], Judith Kikhney [3,4], Annette Moter [3,4,5] and Helena Bujdáková [1]

[1] Department of Microbiology and Virology, Faculty of Natural Sciences, Comenius University in Bratislava, Ilkovičova 6, 842 15 Bratislava, Slovakia
[2] Department of Environmental Health, Research and Development Unit, National Institute of Health Dr Ricardo Jorge (INSA), Av Padre Cruz, 1649-016 Lisboa, Portugal
[3] Biofilmcenter, Institute of Microbiology, Infectious Diseases and Immunology, Charité—Universitätsmedizin Berlin, Hindenburgdamm 30, 12203 Berlin, Germany
[4] MoKi Analytics GmbH, Charité-Universitätsmedizin Berlin, Hindenburdamm 30, 12203 Berlin, Germany
[5] Moter Diagnostics, Marienplatz 9, 12207 Berlin, Germany
* Correspondence: barbora.gaalova@uniba.sk; Tel.: +421-2-9014-9480

Phenotype identification of *Candida albicans*

The standard strain of *Candida albicans* was verified by the growth on CHROMagar Candida (Liofilchem, Italy), cultivated at 48 h at 37 °C. The selective medium was inoculated by a volume of 20 μL of an overnight yeast culture.

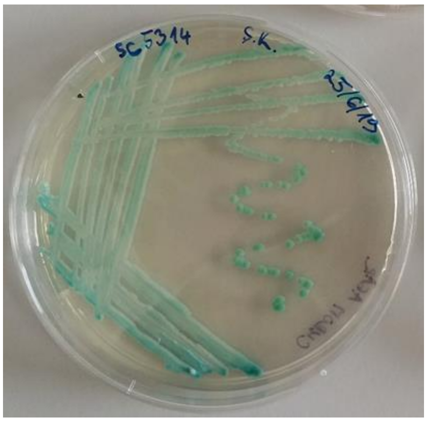

Figure S1. Specific growth of *C. albicans* SC 5314 on CHROMagar Candida.

Phenotype and genotype identification of *Staphylococcus aureus* isolates

The *S. aureus* strains were re-identified using different phenotypic and genotypic methods. For phenotype identification of strains, the Petri dish with Mannitol Salt Agar (Biolife, Italy) was inoculated by a volume of 20 μL of an overnight bacterial culture. After 24 h cultivation at 37 °C, the morphology of colonies and change of the agar color due to the fermentation of mannitol were considered. Beta-hemolysis on Columbia blood agar with 5% of sheep blood (BD, USA) was confirmed in all isolates. Growth curves of each isolate were prepared after adjustment of overnight bacterial culture in Mueller Hinton Broth (MHB) to OD_{600} 0.01 and measured every 30 minutes. The results of phenotype characterization of isolates are shown in **Figure S2.**

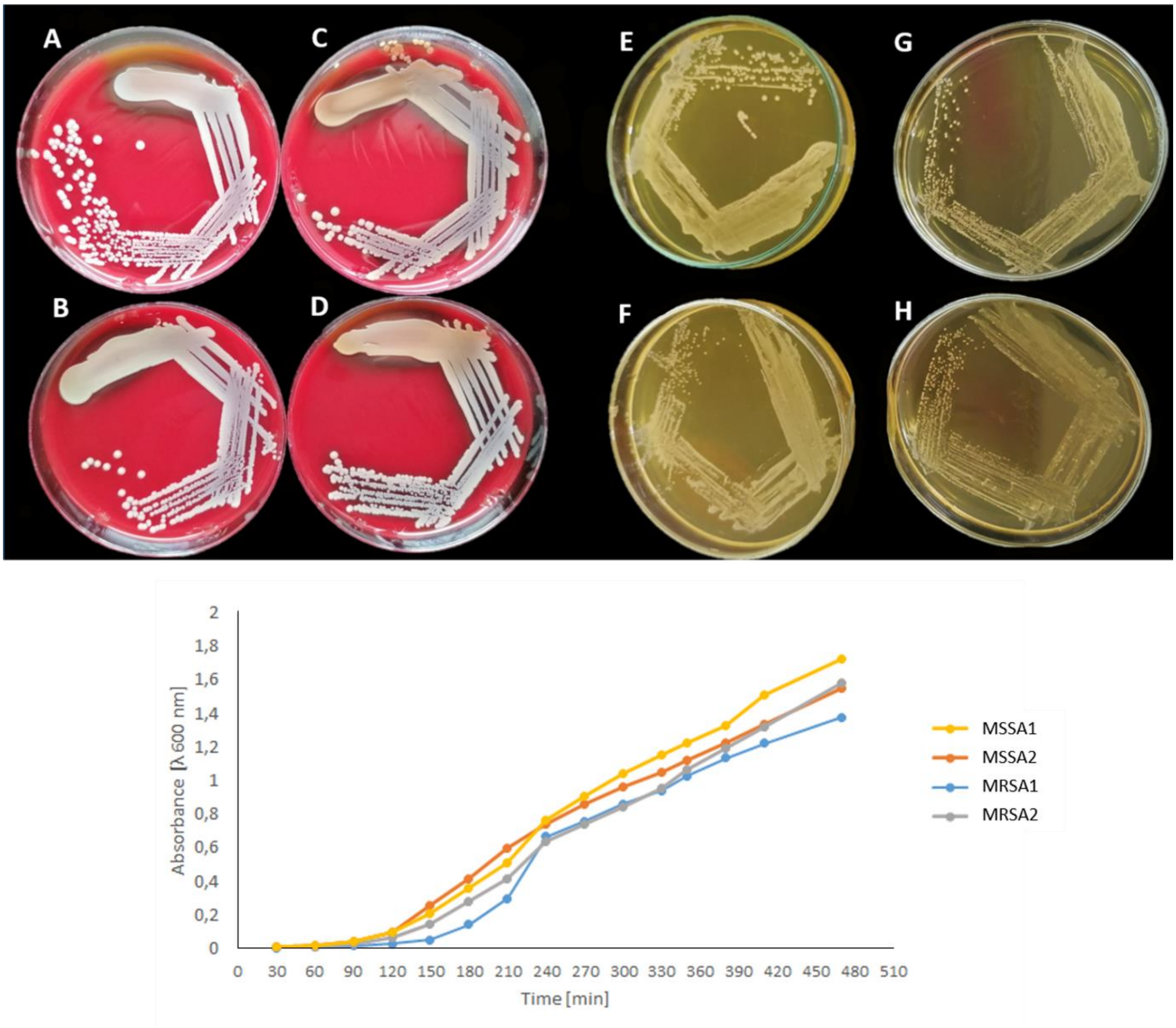

Figure S2. Phenotype identification and growth curves of *S. aureus* isolates: upper image: A) *S. aureus* CCM 3953; B) *S. aureus* DRA; C) *S. aureus* DHN; D) *S. aureus* L18 (Columbia blood agar with 5% of sheep blood); E) *S. aureus* CCM 3953; F) *S. aureus* L18; G) *S. aureus* DRA; H) *S. aureus* DHN (Mannitol Salt Agar). The graph below documents the growth curves of *S. aureus* isolates achieved by measuring of optical density (OD_{600}) of suspension in 30 min intervals.

Genotypic identification of *S. aureus* isolates included polymerase chain reaction (PCR) of species-specific regulator of methicillin resistance (*femA* gene). Isolates were divided according to the presence of *mecA* gene, the key factor of PBP2a production, to methicillin-sensitive and methicillin-resistant. For determination of resistance to fluoroquinolones, efflux pumps coded by *NorA*, *norB* and *norC* genes were monitored. From the group of aminoglycoside modification enzymes, genes of *ant(4')-Ia*, *aph(3')-III* and *aac(6')-aph(2")* were tested. Ribosomal binding site modification, which can be associated with resistance to macrolides, streptograminesB and lincosamides, was monitored by detection of the *ermA*, *ermB* and *ermC* genes (erythromycin ribosome methylases) and efflux pumps *msrA* and *msrB*.

Genomic DNA was isolated with HigherPurity™ Bacterial Genomic DNA Isolation Kit according to the manufacturer's instructions (CanvaxBiotech, Spain). Oligonucleotide primer sequences and their properties are listed below (**Table S1)**.

The total volume of the reaction was 20 μL and consisted of 4 μL 5x FIREPol® Master Mix (Solis BioDyne, Estonia), 1 μL (0.01 – 10 ng/μL) of template DNA, 0.5 μL of 10 pM Forward primer, 0.5 μl of 10 pM Reverse primer and 14 μL Nuclease-free water. The PCR reaction was performed in an iCycler Thermal Cycler (BIORAD, USA). As a negative control, Nuclease-free water was used. The PCR temperature cycling conditions for *femA* were as follows: initial denaturation at 95 °C for 15 min; followed by 34 cycles of denaturation at 95 °C for 20 sec, annealing at 54.7 °C for 1 min, and elongation at 72 °C for 1 min. The final cycle was followed by an extension at 72 °C for 10 min.

For *mecA* the program was the same, only the annealing temperature differed.

Visualization of PCR products was performed in 1.5% agarose gel in 1x Tris borate buffer (TBE) with 4 μL of GoodView Nucleic Acid Stain-HGV-II (SBS Genetech, China) and the DNA Ladder (Invitrogen, USA) was used for estimating the length of products. Electrophoresis was performed in the conditions of 80 V for 90 min (PowerPac™, Bio-Rad Laboratories Inc., USA). After separation, DNA fragments were visualized using an UV-Transilluminator MUV 21-312-220 (Major Science, USA) at a wavelength of 254 nm. Results are shown in the **Figure S3**.

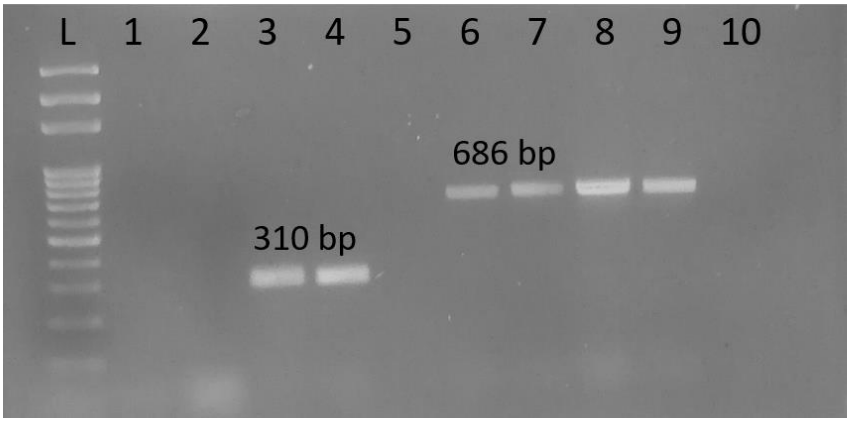

Figure S3. PCR detection of *mecA* and *femA* genes in isolates of *S. aureus*: L – 100- 2000 kb DNA Ladder; 1. - *mecA* MSSA1; 2. - m*ecA* MSSA2; 3. - *mecA* MRSA1; 4. - *mecA* MRSA2; 5. - *mecA* negative control; 6. - *femA* MSSA1; 7. - *femA* MSSA2; 8. - *femA* MRSA1; 9. - *femA* MRSA2; 10. - *femA* negative control.

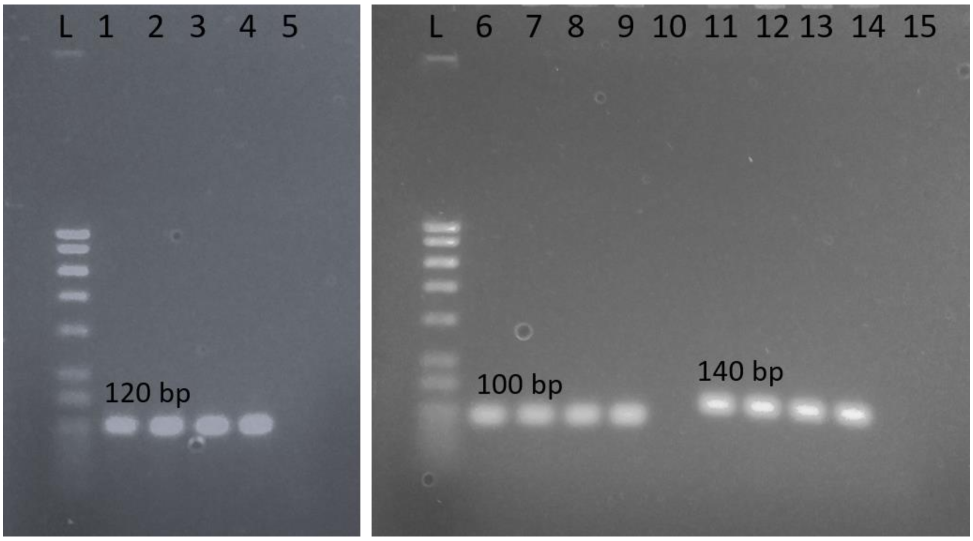

Figure S4. PCR detection of *norA, B, C* genes in isolates of *S. aureus*: L – DNA ladder (25-700 bp); 1. – *norA* MSSA1; 2. – *norA* MSSA2; 3. - *norA* MRSA1; 4. – *norA* MRSA2; 5. – *norA* negative control; 6 - *norB* MSSA1; 7. - *norB* MSSA2; 8. - *norB* MRSA1; 9. - *norB* MRSA2; 10. - *norB* negative control; 11. - *norC* MSSA1; 12. - *norC* MSSA2; 13. - *norC* MRSA1; 14. - *norC* MRSA2; 15. - *norC* negative control.

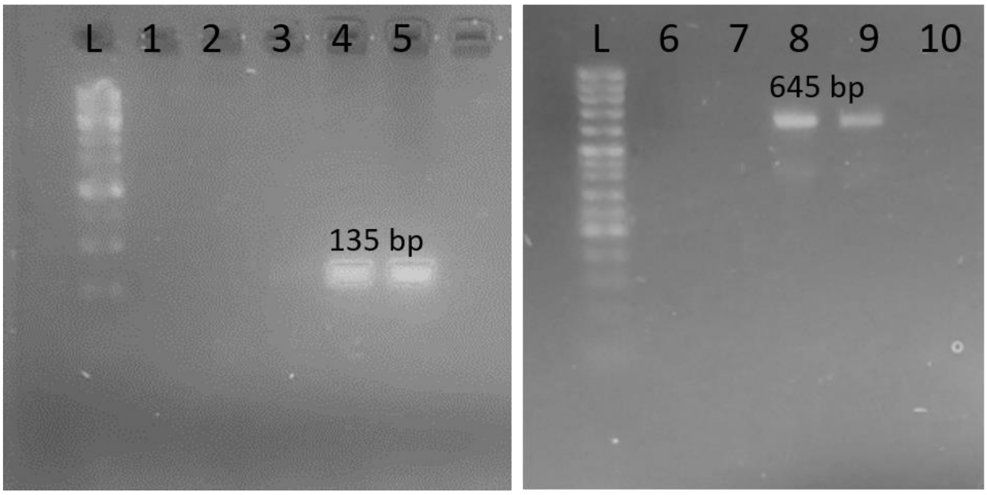

Figure S5. PCR detection of *ant(4′)-Ia* and *ermA* genes in isolates of *S. aureus*: L – 100- 2000 kb DNA Ladder; 1. – *ant(4′)-Ia* negative control; 2. – *ant(4′)-Ia* MSSA1; 3. – *ant(4′)-Ia* MSSA2; 4. *ant(4′)-Ia* MRSA1; 5. – *ant(4′)-Ia* MRSA2; 6 – *ermA* MSSA1; 7 - *ermA* MSSA2; 8 - *ermA* MRSA1; 9 - *ermA* MRSA2; 10 - *ermA* negative control.

Table S1. List of oligonucleotide sequences used in this study. All primers were synthesized by Metabion International AG, Germany.

Gene		Sequence 5′- 3′	Amplicon size	Reference
femA	forward	CTT ACT TAC TGG CTG TAC CTG	686 bp	Vannuffel et al., 1999 [34]
	reverse	ATG TCG CTT GTT ATG TGC		
mecA	forward	GTA GAA ATG ACT GAA CGT CCG ATA A	310 bp	

	reverse	CCA ATT CCA CAT TGT TTC GGT CTA A		Martineau et al., 2000 [72]
norA	forward	TGC CTG GTG TGA CAG GTT TA	120 bp	Kong et al., 2017 [32]
	reverse	AAT CCA CCA ATG CCT GGT CC		
norB	forward	ATG GAA AAG CCG TCA AGA GA	110 bp	Kong et al., 2017 [32]
	reverse	AAC CAA TGA TTG TGC AAA TAG C		
norC	forward	ATG AAT GAA ACG TAT CGC GG	120 bp	Kong et al., 2017 [32]
	reverse	GTC TGC ACC AAA ACT TTG TTG TAA A		
ant(4')-Ia	forward	AAT CGG TAG AAG CCC AA	135 bp	Choi et al., 2003 [73]
	reverse	GCA CCT GCC ATT GCT A		
aph(3')-III	forward	AAA TAC CGC TGC GTA	242 bp	Choi et al., 2003 [73]
	reverse	CAT ACT CTT CCG AGC AA		
aac(6')-aph(2")	forward	GAA GTA CGC AGA AGA GA	491 bp	Choi et al., 2003 [73]
	reverse	ACA TGG CAA GCT CTA GGA		
ermA	forward	TCT AAA AAG CAT GTA AAA GAA	645 bp	Sutcliffe et al., 1996 [74]
	reverse	CTT CGA TAG TTT ATT AAT ATT AGT		
ermB	forward	GAA AAG GTA CTC AAC CAA ATA	639 bp	Sutcliffe et al., 1996 [74]
	reverse	AGT AAC GGT ACT TAA ATT GTT TAC		
ermC	forward	TCA AAA CAT AAT ATA GAT AAA	642 bp	Sutcliffe et al., 1996 [74]
	reverse	GCT AAT ATT GTT TAA ATC GTC AAT		
msrA	forward	GGC ACA ATA AGA GTG TTT AAA GG	939 bp	Ojo et al., 2006 [75]
	reverse	AAG TTA TAT CAT GAA TAG ATT GTC CTG TT		
msrB	forward	TAT GAT ATC CAT AAT AAT TAT CCA ATC	595 bp	Rossato et al., 2020 [76]
	reverse	AAG TTA TAT CAT GAA TAG ATT GTC CTG TT		
